# The manifestation and causes of public panic in the early stage of COVID-19 in China: a framework based on consciousness-attitude-behavior

**DOI:** 10.3389/fpubh.2024.1324382

**Published:** 2024-12-03

**Authors:** Changwei Wei, Jiaxi Xu, Zuying Xu

**Affiliations:** ^1^School of Public Policy and Management, China University of Mining and Technology, Xuzhou, China; ^2^School of Political Science and Public Administration, Wuhan University, Wuhan, China; ^3^School of Economics and Management, Huaibei Normal University, Huaibei, China

**Keywords:** COVID-19, epidemic, panic consciousness, panic attitude, panic behavior

## Abstract

**Background:**

The onset of the COVID-19 pandemic brought about a stark and devastating impact on global scales, affecting countries and their citizens profoundly. The public’s lack of readiness for such an enigmatic and virulent threat led to widespread alarm, catalyzing a paradigm shift in both public conduct and governmental tactics. In the midst of this urgency, there was a notable lack of studies on the initial panic waves. Our study is designed to investigate the dynamics of public panic during the early stages of the pandemic, including its origins, and the public’s perceptions and behaviors.

**Methods:**

Our research, conducted through a questionnaire survey employing snowball sampling, gathered critical data on the public’s awareness, attitudes, and behaviors related to panic between February 23rd and March 25th, 2020.

**Results:**

The findings indicate a period of exceptionally intense and authentic public panic. This panic was a pervasive sentiment, manifesting in strong endorsements for rigorous epidemic control measures and heightened anxiety over virus-related information and family safety. The rapid spread of panic was also a notable characteristic.

**Conclusion:**

The public panic in response to COVID-19 was modulated by stringent prevention measures, with anxiety levels differing significantly based on occupation and health awareness. Notably, the rise of suspicious and distrustful actions was inextricably linked to an overwhelming sense of fear that gripped the public.

## 1 Introduction

The incidence and severity of disasters have been on the rise, posing a grave threat to human life, as well as to social and economic stability (1). Such traumatic events take a toll on the mental well-being of individuals (2). The distressing toll in human lives can profoundly affect public consciousness, tapping into the innate human fear of mortality and potentially triggering widespread group panic. In the grip of extreme fear, members of the public may resort to excessively aggressive actions, thereby intensifying the crisis’s devastating impact (3).

At the end of 2019 and the early of 2020, COVID-19 broke out suddenly. Coincidently with the Chinese Spring Festival, it quickly spread across the whole country (4). The disruption of societal production and the orderly conduct of daily life, along with the endangerment of public safety and property, fueled a growing sense of panic as the disease intensified. In the initial stages of the outbreak, public concern was largely centered on the virus itself, with individuals from Wuhan, Hubei province, and other inter-regional locations becoming the focal points of panic due to their potential to carry the virus (5). The unexpected outbreak of COVID-19 caught the government off guard, resulting in delays in the dissemination of accurate information regarding the origins, transmission, and risks associated with the pandemic (6). At the outset of the crisis, there was chaos in the allocation of essential supplies, leading to varying degrees of shortages in epidemic prevention materials such as medical masks, protective clothing, and thermometers (7). The scarcity of these materials quickly became a significant source of public anxiety. Confronted with the abrupt onset of the epidemic, the Chinese government exhibited certain shortcomings in crisis preparedness, information transparency, organizational response, and post-disaster relief efforts, which exacerbated the proliferation of rumors and, in turn, magnified public panic (8). Moreover, COVID-19 presented a formidable public health challenge to China and numerous other countries, leaving an indelible mark on public health systems, human lives, and the national and global economies (9). This impact was vividly reflected in the spate of business closures, escalating prices, and soaring unemployment rates, which introduced another layer of public panic (10).

In his examination of panic triggers, Becker demonstrated that the overwhelming drive behind human action is the quest for predictability in one’s life, aimed at banishing the uncertainty of the unknown. The inevitability of death, coupled with the uncertainty of its timing and location, instills a profound sense of dread in humans (11). It follows that personal panic is a quintessential emotional response to actual threats to life and property. Indeed, the panic mindset can be understood as a crisis of social trust—a behavioral response to the acute lack of faith individuals experience in their environment during sudden emergencies, and a reflection of society’s collective response to crisis events (12). Personal panic has the potential to cascade into public panic. In their study of panic during emergencies, Başoǧlu and associates identified a link between the emergence of group panic and factors such as group membership, density, and intergroup relationships (13). Le Bon’s insights suggest that individuals within a group are susceptible to the allure of an idea, which, once introduced, can spread rapidly through the group due to its contagious nature. Moreover, individuals in groups often forego independent thought, instead passively adopting the ideas and thoughts of others. Group behavior manifests when individuals within the group become self-aware and actively seek behavioral norms that guide their actions; these norms serve as invaluable compasses in emergency situations and chaotic group settings, aiding individuals in navigating immediate challenges (14). Tald posited that “social mimicry” is a cornerstone of sociology, with the process of societal change being essentially a social interaction—or a form of mimicry. In times of tragedy or crisis, the innate human tendency to worry compels individuals to conform to collective behavior, leading to a phenomenon of excessive conformity (15).

Panic is an innate psychological response triggered when an individual confronts potentially hazardous elements in their environment, and this psychological state exerts a significant influence on human behavior (16). Building upon this understanding, the perspective of this paper is that panic represents both a physiological and social reaction to danger. This reaction not only prompts behavioral changes but also subtly compels individuals to adopt corresponding measures. Such responses are often characterized by irrationality and blindness. The impact of crises on the public engenders a mass panic, a spontaneous reaction of the populace to an unexpected disaster. This subjective and conscious response typically ignites a more widespread, unconscious panic among the public (17). This dynamic often initiates a chain reaction, where individual panic escalates into public panic, which in turn intensifies the severity of individual panic.

The onset of an unexpected public crisis can readily prompt psychological reactions among the populace, such as tension, anxiety, and even panic, which may subsequently result in psychological disorders like stress disorder and depression. The American Psychological Association acknowledges that a moderate level of fear can be beneficial, as it motivates individuals to take prompt action to safeguard themselves against threats like COVID-19 (18). However, when fear becomes excessive, it can precipitate significant medical and psychological challenges, placing both the body and mind under prolonged periods of heightened stress (19). Terror Management Theory (TMT) posits that the fundamental source of individual anxiety is the fear of death, and it emphasizes the need for a scientific approach to managing the anxiety that stems from this “fear of death” (20). Research has revealed that in the face of mortality, individuals often seek stronger social connections to attain a sense of safety and belonging within a group (21). This group belonging, as a cultural worldview, has been found to assist the public in mitigating their fear of death (22). Furthermore, it has been observed that the psychological protection afforded by deep interpersonal bonds can surpass the comforting effects provided by cultural worldviews (23). Furthermore, Coelho et al. have identified that fear of the unknown is a significant catalyst for the anxiety that arises in the face of unpredictable and uncontrollable events, heightening the public’s emphasis on negative emotions and fostering a more acute sense of panic (24). Throughout the COVID-19 pandemic, a crucial correlation has emerged between the degree of uncertainty and the management of information channels. Specifically, it has been observed that panic can be effectively modulated by adjusting the flow of information—either by expanding or constraining the information channels available to the public (25).

Following the outbreak of COVID-19, the pandemic exerted a direct and profound impact on governments at all levels and the general populace (26). Confronted with an unfamiliar and highly transmissible virus, residents found themselves ill-prepared, leading to a pervasive phenomenon of panic (27). This collective anxiety influenced the behaviors of both citizens and the government. A thorough understanding of public panic is essential for comprehending the behavioral responses of residents and for enabling managers to refine their decision-making processes. This study gathered data on the public’s consciousness, attitudes, and behaviors in the wake of the epidemic through a questionnaire survey, aiming to dissect the manifestations and underlying causes of public panic. The data were collected in the aftermath of the first wave of COVID-19 in China, with the survey conducted between February 23 and March 25, 2020. During this period, the public’s panic was particularly palpable and authentic, providing valuable insights into the genuine reactions of the public. This information has been instrumental in analyzing the real-time responses of the populace to the crisis, offering a more nuanced understanding of the dynamics of public panic.

## 2 Theoretical basis

Undoubtedly, public crises will affect many people. In fact, after a public crisis occurs, people in a state of stress experience abnormal changes in their cognition, attitude, and reaction behavior (28). Paying attention to people’s cognition, attitude, and behavior during crises can help to better understand public crises and improve crisis management (29). This article mainly constructs the foundation of analysis based on two theories.

One is knowledge, attitude/belief and practice theory which is also abbreviated as KAP theory. It is the most commonly used model to explain how an individual’s knowledge and attitudes beliefs influence changes in healthy behavior. At present, this theory has been widely used in various fields such as community chronic disease management, nursing education, nursing management, and health education, and has achieved significant results (30). The KAP theory divides changes in human behavior into three continuous processes: acquiring knowledge, generating attitudes/beliefs, and forming behavior (31). Among them, “K” refers to the recognition and understanding of relevant knowledge, “B” refers to correct beliefs and positive attitudes, and “P” refers to actions. The KAP theory proposes a progressive relationship between knowledge, attitudes, and behavior. Knowledge is the foundation of behavioral change, while beliefs and attitudes are the driving forces behind behavioral change (32). According to this theory, it is inferred that individuals must first acquire relevant knowledge and then develop positive perceptions thereof to gradually cultivate beliefs. Moreover, it is the transformation of knowledge into belief that paves the way for positive attitudes, ultimately leading to behavioral change.

The second theoretical construct examined is the Affective-Behavioral-Cognitive (ABC) theory, a concept formulated by Albert Ellis that has become deeply ingrained in the field of psychology. This theory primarily addresses the interplay between human behavior and emotions, offering insights into understanding the behavioral and emotional responses of individuals (33). The ABC theory is underpinned by a cognitive mediation model, where “A” represents activating events or experiences—those events, activities, or agents that provoke disturbance in individuals. “B” denotes the rational or realistic beliefs that individuals hold regarding these activating events, which are likely to result in “C,” the consequences—both behavioral and emotional—of the interaction between A and B (34). According to this theory, different people may react differently to the same event, a discrepancy that is attributed to their individual beliefs. These beliefs are shaped by a myriad of factors, including culture, education, and personality (35). This theory has also been introduced by some scholars into the study of human behavior and emotional responses in crisis situations. Mayer and his collaborators raised three-dimensional model for crisis assessment or intervention. This theory assesses affective, behavioral, and cognitive reactions of people to crisis events (36). They evaluated three main emotions of people during the crisis, three cognitions in four areas of life, and three types of behavioral responses (37).

In alignment with the aforementioned theories, when confronted with sudden crisis stimuli, individuals exhibit corresponding responses across cognitive, attitudinal, and behavioral domains. An individual’s perception of the crisis—comprising factors such as the availability of information, perceived controllability, and the anticipated duration—shapes their attitudes and beliefs (38). The interplay between one’s perception and attitude toward crises is ultimately reflected in their behavioral and emotional reactions. This conceptual framework has been utilized to examine and understand the psychological and behavioral states of individuals and groups in various public health events, including the SARS. For instance, the study conducted by Dorfan and Woody demonstrated a significant correlation between individuals’ assessments of the SARS and their emotional and behavioral responses (39). Other research has indicated that knowledge and perceptions of SARS are linked to preventive behaviors. Similarly, this approach has been extended to the study of individuals’ behaviors and psychology in the context of COVID-19. For example, Xu and colleagues conducted an online cross-sectional survey of the Chinese population to examine the associations between COVID-19 infection concerns, public risk perception, information sources, knowledge, attitudes, and behaviors (40). Theoretically, we can dissect the public’s panic responses in the early stages of COVID-19 through an analysis of the three dimensions of consciousness, attitude, and behavior.

## 3 Methods

### 3.1 Questionnaire design

This study employed a questionnaire survey to investigate the public’s panic consciousness, attitudes and behavior toward COVID-19. From the perspective of virus nature and transmission characteristics, COVID-19 is very similar to SARS many years ago. Therefore, when designing the questionnaire, we mainly referred to the survey questionnaire on public panic during SARS. Moreover, these questionnaires have become relatively mature and have been successfully used in survey practice. This questionnaire is based on the SARS stress response questionnaire developed by Professor Huge Tone of Soochow University and the public risk awareness and psychological behavior survey questionnaire developed by Professor Huaibin Jing et al. of Sun Yat sen University, combined with the public health part of the public safety survey questionnaire conducted by China University of Mining and Technology for many years. Tone’s questionnaire consists of 13 questions, covering three aspects: cognitive evaluation of SARS, panic about SARS, and defensive psychology and behavioral responses to SARS. The measurement tool professor Jing used includes two questionnaires. The first questionnaire measures the negative psychological reactions caused by SARS, mainly including 10 questions related to self-evaluation of mental stress, depression, anxiety, and trauma. The second questionnaire measures the influencing factors of an individual’s response to SARS, mainly involving aspects such as personality, environment, and individual beliefs.

Drawing upon established theories and leveraging existing, well-honed questionnaires, we have crafted a practical questionnaire to rapidly assess individual panic phenomena in the wake of the first wave of COVID-19 in China. The survey questions were tailored to gauge the impact of COVID-19 on various aspects of daily life, including individuals’ understanding, perceived controllability, and the threat posed to life and property. The questionnaire is structured into four main sections:

The initial section captures demographic factors, encompassing gender, age, education level, occupation, and monthly income. It also includes three specific individual influencers: presence of relatives or friends in Hubei, suspected or confirmed cases in the residential area, and the current residential location.

The second section delves into individual panic consciousness, comprising five items designed to assess the extent of fear regarding COVID-19, the anticipated duration of the pandemic, fear of returning from Hubei, fear of infection, and the overall impact of COVID-19 on life. With the exception of the duration item, these use a 5-point Likert scale, ranging from “1” for “strongly agree” to “5” for “strongly disagree.”

The third section measures individuals’ attitudes toward COVID-19 and associated prevention and control measures, covering six items related to information comprehension, transparency, the perceived controllability of the epidemic, threats to life and income, attitudes toward lockdown measures, and satisfaction with epidemic prevention and control efforts. Responses are again recorded using the 5-point Likert scale.

The final section aims to capture key behavioral responses to the threat of COVID-19, with four items utilizing the 5-point Likert scale. These items focus on behaviors such as stockpiling masks, purchasing essential medications, self-isolating at home, and disseminating information to relatives and friends about COVID-19. Additionally, the survey includes further questions to gain insights into individuals’ behavioral reactions, such as mask usage, daily dedication to following epidemic news, compliance with reporting COVID-19 symptoms, reasons for non-compliance, and whether indifferent behavior toward COVID-19 was promptly corrected.

Overall, the panic consciousness questions are crafted to reflect public anxiety and concern, while the panic attitude questions aim to discern public perceptions of COVID-19. The examination of panic behaviors seeks to visually capture the public’s responses to COVID-19 and analyze whether these responses are logically sound and objective.

### 3.2 Investigation implementation

**Figure 1:**
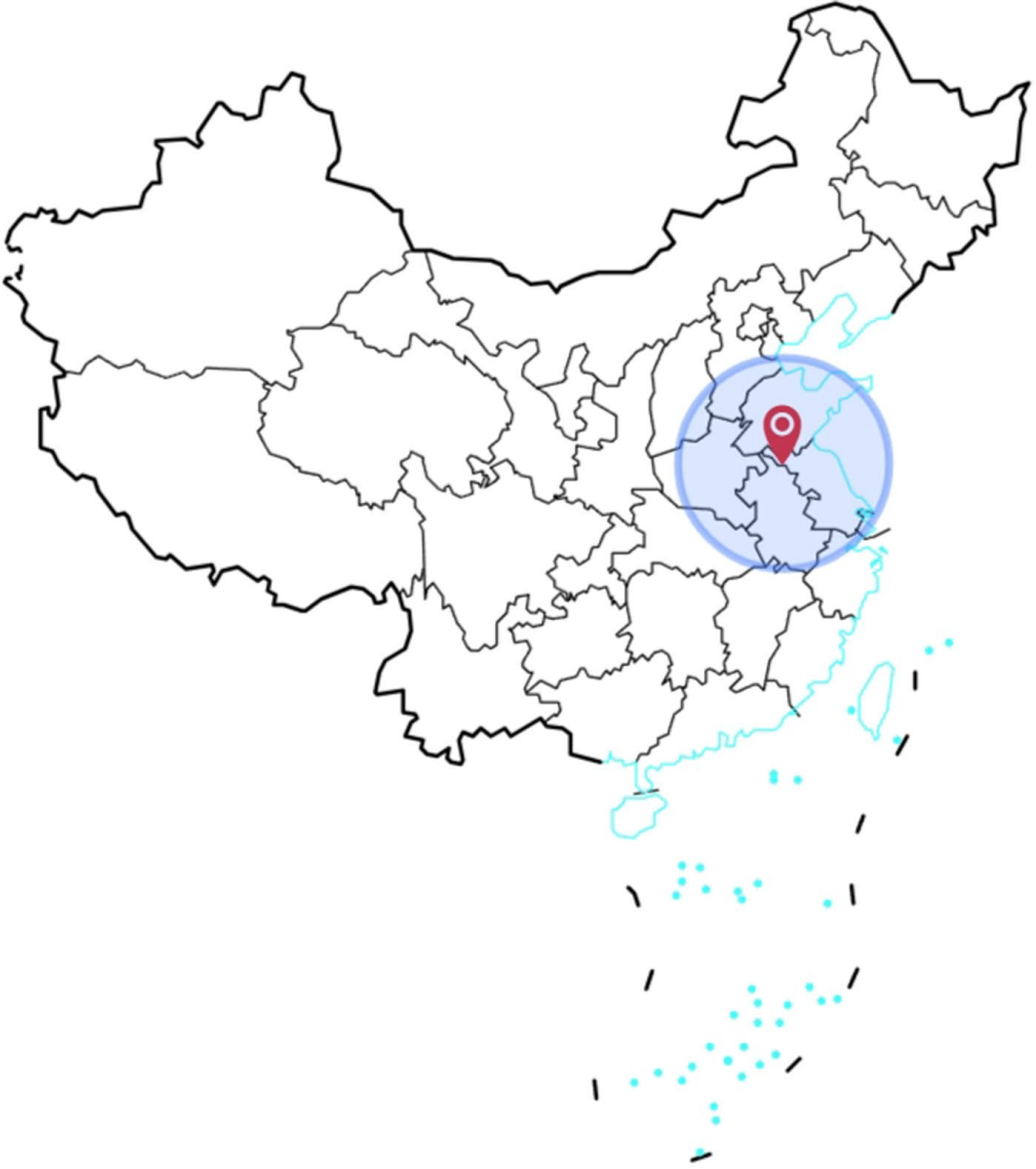
Region in which the research was conducted (location in China).

**Figure 2:**
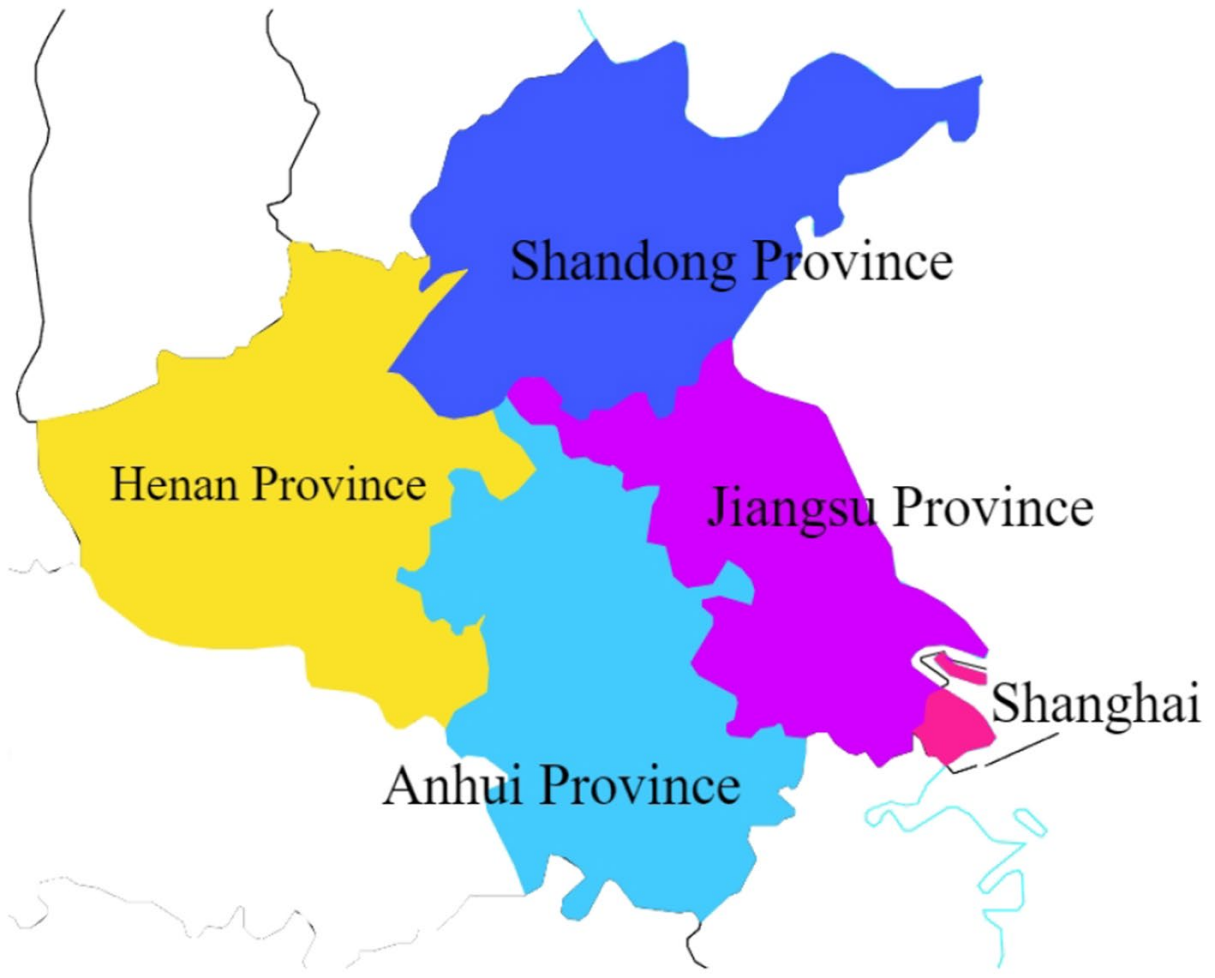
Region in which the research was conducted (specific to province and city).

The survey was carried out between February 23 and March 25, 2020, a period marked by extensive epidemic prevention efforts across China in response to the COVID-19 outbreak. The pandemic’s reach was universal, instilling negative emotions such as anxiety, panic, and discomfort in nearly every Chinese citizen. In the wake of the outbreak, public reactions were widespread and palpable. As part of our urban public safety research project, funded by the National Social Science Foundation of China, we promptly initiated a questionnaire survey to explore this phenomenon. In compliance with the epidemic prevention policies in effect at the time, we executed an online peer-to-peer survey. Utilizing the Chinese questionnaire platform, Questionnaire Star, we distributed the questionnaires to anonymous individuals across several Chinese cities. To maximize participation, we adopted the snowball sampling method (41, 42), leveraging personal social networks to disseminate the questionnaires recursively. The research conducted focuses on the area surrounding Xuzhou City, Jiangsu Province, which was selected as the study’s epicenter due to its strategic significance as the base of the authors’ affiliated research institution. The city’s robust transportation network and its status as a crossroads of multiple provincial boundaries enhance research feasibility and accessibility. Given our extensive social networks within Jiangsu Province, as well as in the adjacent regions of Shanghai, Anhui Province, Shandong Province, and Henan Province, the questionnaires were primarily disseminated among respondents in these areas to leverage local connections and ensure a comprehensive data collection process (Figures 1 and 2).

The entire survey process was conducted with strict adherence to the standardized procedures and ethical guidelines governing online research. The decision to distribute the questionnaires within urban areas was based on the premise that city dwellers are more likely to be internet-savvy and engaged with current affairs. Furthermore, it was assumed that a majority of urban residents hold stable employment and have ready access to mobile devices or computers, thereby facilitating their ability to participate in the survey. These considerations were in harmony with the objectives and requirements of our study. The questionnaires were distributed and collected on an anonymous basis, with participants receiving no financial incentives, thereby ensuring that the data gathered was as unbiased and objective as possible. Despite the final sample size being less than optimal, our rigorous research methodology and subsequent screening of the questionnaires ensured a satisfactory level of diversity among respondents. In the context of the COVID-19 epidemic, public attention was largely directed toward personal safety and adapting to significant changes in lifestyle and work routines. This focus necessarily influenced the number of questionnaires that could be collected, presenting an objective challenge to the survey’s reach.

This survey research embarked on its journey 4 years prior, characterized by its longitudinal and intricate design, necessitating a substantial period for the thorough collection and meticulous analysis of data. To guarantee the precision and dependability of our findings, we embarked on multiple iterations of validation and refinement post-data analysis. Following this comprehensive process, the research entered a stringent peer-review phase, which, albeit critical for maintaining quality, introduced additional delays in the publication timeline. We extend our heartfelt gratitude to all participants for their patience and invaluable contributions. We are confident that the meticulously prepared and rigorously validated results presented in this study will offer significant insights and advancements for both research and practical applications in the field.

### 3.3 Investigation results

A total of 402 surveys were gathered, with 376 valid questionnaires for a 93.53% validity rate. Our main reason for excluding invalid questionnaires is to consider the following points. Firstly, the respondents did not complete the questionnaire, we gave up questionnaires with more than five unanswered questions. Secondly, participants were inclined to respond to the items in a similar pattern, for example, too many results were concentrated in one value. Thirdly, it is not a peer-to-peer questionnaire collection. Fourthly, eliminating questionnaires collected after the deadline. Finally, excluding questionnaires that do not meet age requirements.

Respondents were mainly concentrated in the central and eastern regions of China (which also relates to the city in which the authors’ research organization is located). The demographic characteristics of the respondents are shown in Table 1. 42.29% of male residents and 57.71% of female residents were interviewed. In terms of age, 16.49% of the population is over 50, 26.33% is 36–50 years old, 12.5% is 26–35 years old, 44.68% is 18–25 years old. It is important to note that, despite the peer-to-peer questionnaire design, the online format resulted in a small number of respondents under the age of 18. As they did not meet the target audience requirements of the study, they will be excluded from subsequent analyses. The results of this research collection comprise 28 persons with junior college degrees (7.45%), 151 people with bachelor’s degrees (40.16%), and 44 people with graduate degrees and above (11.7%). The total number of people with higher education is 223, accounting for 59.31% of the total. “Do you have friends or relatives who are from Hubei or live and work in Wuhan?” In this item, 82 people, or 21.81 percent, explicitly said yes, while 277 people, or 73.67 percent, explicitly said no. Comparatively speaking, the proportion of those who have friends and relatives in Hubei or Wuhan is still relatively high. As many as 64 people, or 17.02% of those polled, claimed unequivocally that there were suspected or confirmed cases in their communities. However, as many as 42 respondents stated they were unsure and did not know, accounting for a startling 11.17 percent of the total.

**Table 1:** Demographics characteristics of the study sample (*n* = 376).

Variable	Category	Number of respondents	Percent of respondents
Sex	Female	159	42.29%
	Male	217	57.71%
Age	18–25 years	168	44.68%
	26–35 years	47	12.5%
	36–50 years	99	26.33%
	>50 years	62	16.49%
Education	Junior high school and below	60	15.96%
	Senior high school	93	24.73%
	Junior college	28	7.45%
	Undergraduate	151	40.16%
	Postgraduate or above	44	11.7%
Occupation	Enterprise employees	170	45.21%
	Civil servant	53	14.1%
	Medical worker	39	10.37%
	Personnel of public institutions	28	7.45%
	College students	35	9.31%
	Self-employment	22	5.85%
	Retiree	25	6.65%
	Other	17	4.52%
Monthly income	<2000 RMB	38	10.11%
	2001–3,500 RMB	37	9.84%
	3,501–5,000 RMB	196	52.13%
	5,001–8,000 RMB	88	23.4%
	8,001–12,000 RMB	9	2.39%
	>12,000 RMB	8	2.13%
Relatives or friends in Hubei	Yes	82	21.81%
	No	277	73.67%
	Unknow	17	4.52%
Suspected or confirmed cases in the residential area	Yes	64	17.02%
	No	270	71.81%
	Unknow	42	11.17%
Current residential location	Districts of municipalities directly under the central government	34	9.04%
	District of provincial capital city	40	10.64%
	District of prefecture level cities	161	42.82%
	District of county-level cities	67	17.82%
	Township Resident	30	7.98%
	Rural area	42	11.17%
	Other	2	0.53%

## 4 Data analysis

### 4.1 Reliability analysis

The reliability analysis results of this questionnaire survey are shown in Tables 2, 3. The first section of the questionnaire is about public’ panic consciousness, it is intended to understand the general public’s intuitive panic feelings about COVID-19. It has four questions on a 5-point Likert scale. Table 2 displays the initial credibility and corrected reliability. With a Cronbach’s Alpha value of 0.736, which is larger than 0.7, this scale is considered to be reliable. The correlation result of C4 question “I’m very worried about being infected with COVID-19?” is 0.279 in Table 3. It is less than 0.3, indicating weak correlation and that item should be eliminated.

**Table 2:** The credibility of panic consciousness, attitude, and behavior.

Reliability statistic		
Cronbach’s alpha		No. of items
panic consciousness	.660 (initial credibility)	4
	.736 (final credibility)	3
panic attitude	.587 (initial credibility)	6
	.660 (final credibility)	4
panic behavior	.345 (initial credibility)	4

**Table 3:** Total statistics for panic consciousness, attitude, and behavior.

Item	Mean value of the deleted scale of the item	Deleted scale variance of the item	Corrected item-total correlation	Cronbach’s alpha if item deleted
C1: The epidemic has made me feel panic	8.430	7.478	0.580	0.495
C3: I’m very worried about people returning from Hubei around me	8.980	7.765	0.494	0.555
C4: I’m very worried about being infected with COVID-19	7.530	9.247	0.279	0.736
C5: The epidemic has had a great impact on my life	8.870	8.300	0.429	0.601
A1: I have a lot of knowledge about this epidemic	18.730	10.674	0.257	0.569
A2: I think the epidemic is controllable	18.690	9.611	0.419	0.501
A3: The epidemic poses a threat to life and property	18.670	11.362	0.090	0.645
A4: I support implementing closed-off management	18.170	10.217	0.322	0.543
A5: Information about the epidemic is transparent	18.780	9.692	0.393	0.512
A6: The community has been very successful in epidemic prevention and control	18.500	9.010	0.507	0.458
B1: Should try to stock up on masks as much as possible	5.912	4.578	0.169	0.301
B2: Should rush to purchase potentially useful drugs	6.840	4.477	0.343	0.114
B4: Isolation is an effective way to avoid transmission	6.712	4.756	0.069	0.441
B8: Will promote real-time information to family and friends	6.776	5.206	0.201	0.271

The second section of the questionnaire questions is on the credibility research of panic attitude, and it has six questions on a 5-point Likert scale. Because the corrected term total correlations for A1 and A3 were 0.257 and 0.090, respectively, both less than 0.3, so these two terms were discarded. Table 4 displays the adjusted results. The corrected Cronbach’s alpha coefficient value is 0.660, which is less than 0.7 but larger than 0.6. The scale must be corrected again, but the result is still relevant, the data can still be used as a reference for the performance of the public’s attitude toward panic.

**Table 4:** Cross-tabulation of gender and feeling panic or not.

Question	Options	Genders				Total	χ^2^	*p*
		Male	Female	3	4			
The epidemic has made me feel panic	Extreme panic	24(16.11)	48(21.43)	1	0	73(19.41)	19.85	0.07
	Quite panic	20(13.42)	52(23.21)	0	1	73(19.41)		
	Average	48(32.21)	66(29.46)	0	0	114(30.32)		
	Less panic	32(21.48)	40(17.86)	1	0	73(19.41)		
	No panic at all	25(16.78)	18(8.04)	0	0	43(11.44)		
Total		149	224	2	1	376		

*p < 0.05; **p < 0.01.

The third section of the questionnaire questions is on the credibility research of panic behavior, and it has four questions on a 5-point Likert scale. As presented in Table 3, the correlation results for all three items are below 0.3, the correlation result of B2 is only slightly higher than 0.3. Moreover, the Cronbach’s Alpha value of panic behavior is 0.345, indicating weak correlation. So, we do not intend to use these four items for further analysis.

### 4.2 Descriptive analysis

The panic attitude was assigned secondary relevance due to the comparatively low reliability of the panicky attitudes produced from the reliability study. The second component is the public’s perception of how long COVID-19 will last (Table 5).

**Table 5:** How long do you think COVID-19 will last?

	Frequency	Percentage (%)	Effective percentage (%)	Cumulative percentage (%)
Within half a month	43	11.4	11.4	11.4
Half a month ~1 month	77	20.5	20.5	31.9
1 month	75	19.9	19.9	51.9
1 month~2 months	90	23.9	23.9	75.8
2 months~3 months	51	13.6	13.6	89.4
3 months ~ half a year	23	6.1	6.1	95.5
More than half a year	17	4.5	4.5	100.0
Total	376	100.0	100.0	

Approximately 68.1% of the general population believed that the prevention and control of this COVID-19 epidemic will last at least 1 month. It can be observed that most respondents believed that the outbreak will continue for a longer period. This outbreak had a higher impact on the lives of 55.9% of the general public (Table 6), and more than half of the respondents stated that this COVID-19 outbreak had a greater impact on their lives.

**Table 6:** The significant impact of COVID-19 on daily life.

	Frequency	Percentage (%)	Effective percentage (%)	Cumulative percentage (%)
Very large	122	32.4	32.4	32.4
Comparatively large	88	23.4	23.4	55.9
Average	89	23.7	23.7	79.5
Smaller	46	12.2	12.2	91.8
Very small	31	8.2	8.2	100.0
Total	376	100.0	100.0	

In response to the question “If your friends and family members did not care about COVID-19, would you discourage them from doing so?” The proportion of respondents who replied “do not know” and “no” was approximately 13%. The most visible indication of this apathy is their indifference to COVID-19 among their friends and relatives.

According to respondents’ attitudes regarding symptoms such as fever, diarrhea, and colds, 80% of people will take the initiative to report their symptoms as soon as possible, demonstrating that the government’s preventative and control programs have had a more beneficial influence. However, over 20% of responders continue to take medication and experience fever, diarrhea, and cold symptoms without voluntarily reporting to the government.

### 4.3 Correlation analysis

The gender difference on whether or not there was fear about COVID-19 outbreak was investigated using cross-tabulation analysis. Table 4 summarizes the findings.

The results from Table 4 demonstrate that the *p*-value is 0.07 and *p* > 0.05, indicating that gender has no bearing on whether or not they feel panic. There is no statistically significant difference between males and females in their fear feeling of COVID-19.

An ANOVA was used to examine the link between “having relatives or friends in Hubei and worrying about people from Hubei around them” to generate Table 7. The chi-square test was used to investigate the link between “having relatives or friends in Hubei” and “worrying about people from Hubei around them.” The chi-square test was used to study the link between “having relatives or friends in Hubei” and “being concerned about Hubei residents around me.” Since the *p*-value is 0.001 and *p* < 0.05, they are considered to show a significant difference. And since χ^2^ = 38.337, *p* = 0.001 < 0.01, they present a 0.01 level of significance.

**Table 7:** ANOVA on the correlation between relatives or friends in Hubei and worrying about people from Hubei.

Q	Options	Do you have relatives or friends in Hubei			Total	χ^2^	*p*
		Yes	No	Unknow			
C3	Very worried	14(18.42)	123(44.57)	11(52.38)	148(39.36)	38.337	0.001
	Quite worried	25(32.89)	48(17.39)	4(14.29)	77(20.48)		
	Fairly	24(31.58)	46(16.67)	4(14.29)	74(19.68)		
	Not at all worried	11(14.47)	33(11.96)	4(14.29)	48(12.77)		
	Not at all	2(2.63)	26(9.42)	1(4.76)	29(7.71)		
	Total	76	276	24	376		

*p < 0.05; **p < 0.01.

When the percentage differences are compared, it is clear that 52.38% of those who select “no idea” select “very worried,” which is much higher than the average level of 39.36%. The proportion of people who chose “no” and “very worried” is 44.57%, which is much higher than the average of 39.36%. The proportion of people who chose “quite worried” is 50.00%, which is much higher than the average of 20.48%. The proportion of people who select “yes” and “quite worried” is 32.89%, which is much higher than the national average of 20.48%. The proportion of persons who select “yes” above “fairly” is 31.58%, which is much higher than the 19.68% average. As a result of this part, it was discovered that “having relatives or friends in Hubei” has a substantial effect on “worrying about people from Hubei around them.”

The link between “education level” and “occupation” as dependent variables and the dependent variable “how long do you spend paying attention to epidemic information every day” was analyzed using linear regression analysis, and Table 8 was generated.

**Table 8:** Linear regression analysis of the correlation between education level and occupation and the time spent on COVID-19 information.

	Unstandardized coefficient		Standardized coefficient	*t*	*p*	VIF	*R* ^2^	Adjusted *R*^2^	*F*
	B	Std. Error	Beta						
(Constant)	1.719	0.332	–	5.182	0.000**	–	0.1	0.095	20.711 *P* = 0.000
Education level (E)	0.009	0.075	0.008	0.117	0.907	1.9			
Occupation (O)	0.117	0.024	0.321	4.786	0.000**	1.9			

Implicit variable: time spent on COVID-19 information (TSI); D-W value: 1.973; *p < 0.05; **p < 0.01.

From the table above, a linear regression analysis using education level and occupation as independent variables and how long it takes to pay attention to COVID-19 as the dependent variable yields a value of 0.1 for the model R^2^. This implies that at least one part of “education level” and “occupation” will have an effect on the amount of time spent each day on paying attention to this pandemic.

An *F*-test of the model found that the model passed the *F*-test (*F* = 20.711, *p* = 0.000 < 0.05). This suggests that at least one of education level and occupation will have an influential relationship on the amount of time spent on following COVID-19 information daily (abbreviated as TSI). The model equation TSI = 1.719 + 0.009E + 0.117O was derived. In addition, the model was tested for multiple covariances and found that the VIF values in the model were all less than 5, implying that there was no covariance; and the D-W values were around the number 2, thus indicating that there was no autocorrelation in the model and that there was no correlation between the sample data, and that the model was relatively good. The model is relatively good.

Further analysis shows that the regression coefficient value of variable E is 0.009 (*t* = 0.117, *p* = 0.907 > 0.05), which means that variable E does not affect TSI. While the regression coefficient value of variable O is 0.117 (*t* = 4.786, *p* = 0.000 < 0.01), implying that variable O will have a significant positive effect on TSI.

The survey found that “education level” does not have an impact on the time spent on monitoring epidemic news trends every day, while “occupation” has a significant impact on the time spent on monitoring the epidemic. Using a two-factor analysis, “education level” and “occupation” are taken as the major factors, and the location of each individual’s residential community (abbreviated as CRL) is taken as an auxiliary element, to investigate their impact on panic awareness of individuals who feel the impact of COVID-19 on their lives (C5 in Table 3). Table 9 contains the pertinent results.

**Table 9:** Two-way analysis of “education level,” “occupation,” and C5.

Source of variation	Square sum	df	Mean square	*F*	*p*
Intercept	71.415	1	71.415	46.516	0.000**
Education level (E)	8.128	4	2.032	1.323	0.261
Occupation (O)	38.534	10	3.853	2.51	0.006**
Current residential location (CRL)	0.876	1	0.876	0.571	0.45
Residual	552.695	360	1.535		

*R*^2^ = 0.098; *p < 0.05; **p < 0.01.

A two-way analysis of variance (ANOVA) was used to examine the relationship between “education level” and “occupation” on the personal perception of the impact of COVID-19 on life(C5), and community location was included as a covariate in the model. Including community location as a covariate in the model, it can be seen that there is no significance of “education level” (*F* = 1.323, *p* = 0.261 > 0.05), which indicates that “education level” does not have a differential relationship on the C5. Produces a differential relationship. “Occupation,” on the other hand, showed significance (*F* = 2.510, *p* = 0.006 < 0.05), indicating that “Occupation” would have a differential relationship on C5.

In response to research on the factors influencing the public’s satisfaction with epidemic prevention and control in their community, this article used multi-way ANOVA to investigate the impact of “current residential location,” “occupation,” “education level,” and “monthly income” on this. Table 10 summarizes the findings.

**Table 10:** Multivariate analysis of public’s satisfaction with community epidemic prevention and control.

	Square sum	df	Mean square	*F*	*p*
Intercept	134.057	1	134.057	121.742	0.000**
Current residential location (CRL)	8.414	6	1.402	1.273	0.269
Occupation (O)	9.12	10	0.912	0.828	0.602
Education level (E)	17.259	4	4.315	3.918	0.004**
Monthly income (MI)	1.537	5	0.307	0.279	0.924
Residual	385.405	350	1.101		

*R*^2^ = 0.105; *p < 0.05; **p < 0.01.

The R-squared value of the model is 0.105 when using multi-way ANOVA to study the differential relationship between “current residential location,” “occupation,” “education level,” and “monthly income” in terms of “public’s satisfaction with community epidemic prevention and control.” This means that “current residential location,” “occupation,” “education level,” and “monthly income” can explain the 10.54% change in “public’s satisfaction with community epidemic prevention and control.” Furthermore, *p* = 0.000 < 0.01 indicates that “education level” will have a significant difference in satisfaction with community epidemic prevention and control, whereas “current residential location,” “occupation,” and “monthly income” will not have a difference in people’s satisfaction with community epidemic prevention and control.

Meanwhile, based on the survey results, we can further analyze the correlation between public panic and information and uncertainty. As shown in Table 11, we use C1 to test public panic, use C4 and A2 to examine uncertainty, use A1 and A5 to analyze the public’s understanding of relevant information during the epidemic. As we can see from Table 11, although the public’s panic after the first wave of the epidemic was only at an average level (mean value = 2.845), the uncertainty brought about by the epidemic and the transparency of relevant information are troubling the public (the statistical results of C4, A1, A2, and A5 are all much higher than 3). Due to concerns about infection and difficulty in controlling the epidemic, it has clearly led to public panic. The lack of information about the epidemic among the public only has a relatively weak impact on the formation of panic. However, the impact of transparency in epidemic information was not reflected in this survey.

**Table 11:** Analysis of the correlation between panic, information, and uncertainty.

	C1	C4	A1	A2	A5
C1	1	0.374^**^	0.113^*^	0.314^**^	−0.011
C4	0.374^**^	1	0.192^**^	0.371^**^	0.139^**^
A1	0.113^*^	0.192^**^	1	0.244^**^	0.089
A2	0.314^**^	0.371^**^	0.244^**^	1	0.173^**^
A5	−0.011	0.139^**^	0.089	0.173^**^	1
Mean value	2.845	3.736	3.579	3.611	4.133
Standard deviation	1.263	1.288	1.020	1.048	1.036

*p < 0.05; **p < 0.01 C1, The epidemic has made me feel panic; C4, I’m very worried about being infected with COVID-19; A1, I have a lot of knowledge about this epidemic; A2, I think the epidemic is controllable; A5, Information about the epidemic is transparent.

## 5 Results

### 5.1 The manifestations of panic consciousness

Panic consciousness was widely existed in the public, male and female do not show significant gender differences; according to the data analysis of the research results, the cross-analysis of gender and whether to feel panic gets χ^2^-value of 19.85, *p*-value of 0.07, it is obvious that the *p*-value is greater than 0.05. Therefore, according to the results of the cross-analysis, it can be concluded that in term of panic consciousness, there is no significant difference between male and female.

Emotional ties can effectively reduce the public’s sense of panic. By analyzing the ANOVA of “whether there are relatives or friends in Hubei” and “whether they are worried about people coming back from Hubei around them,” we obtained a χ^2^-value of 38.337, and a *p*-value of 0.001, which is not only smaller than 0.05 but also much smaller than 0.01. The respondents of “whether there are relatives or friends in Hubei” are more likely to be worried about people coming back from Hubei around them. This result is not only smaller than 0.05 but also much smaller than 0.01. Respondents of “whether they have relatives or friends in Hubei” show significant differences in all five options of whether they are worried about the people around them coming back from Hubei.

### 5.2 The manifestations of panic attitudes

**Figure 3:**
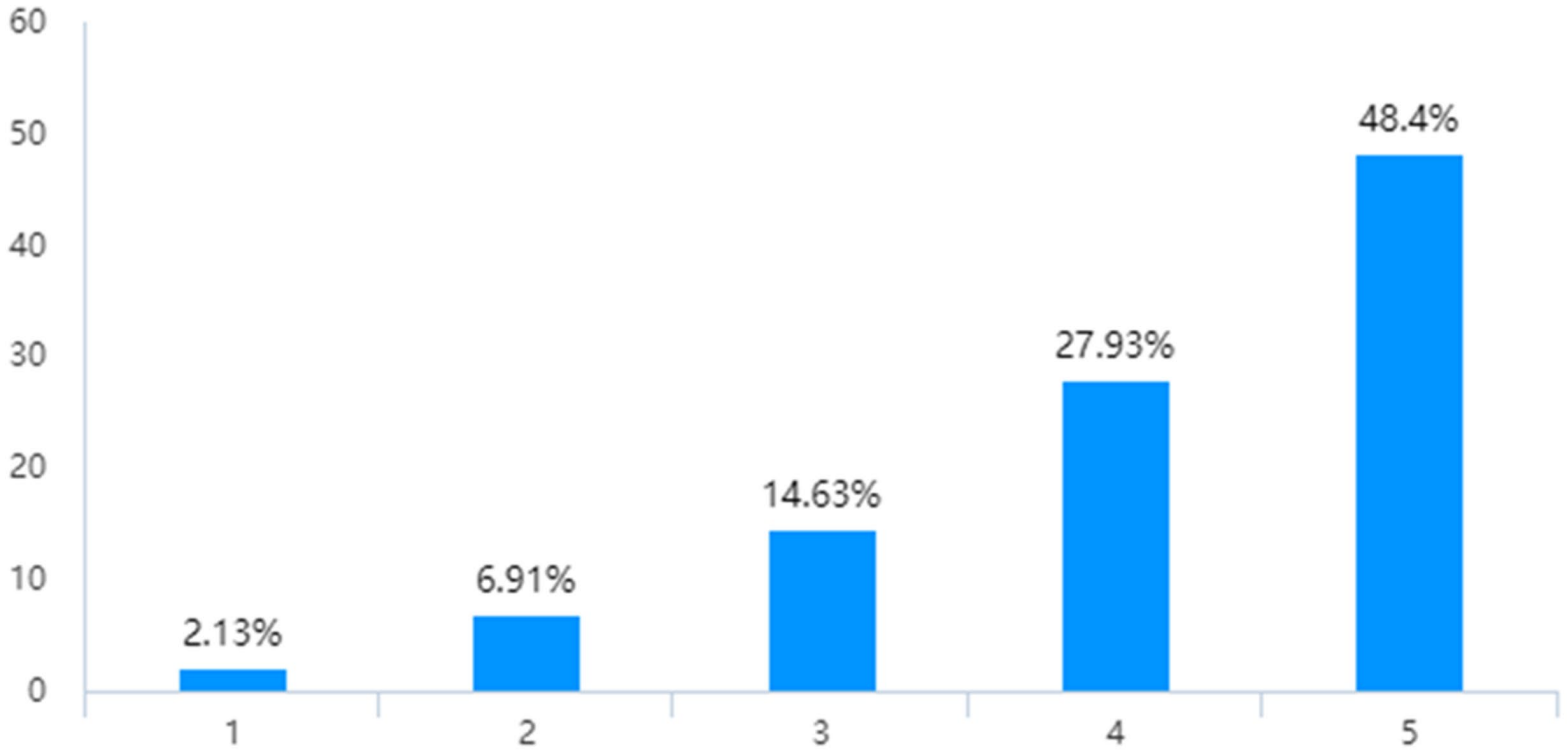
Attitude toward close-off management.

During that special period, the majority of the public supported closed-off management. According to Figure 3, 76.33% of individuals supported the establishment of closed-off management for both neighborhoods and units. The public were generally in favor of rigorous centralized and standardized management throughout the special period.

**Figure 4:**
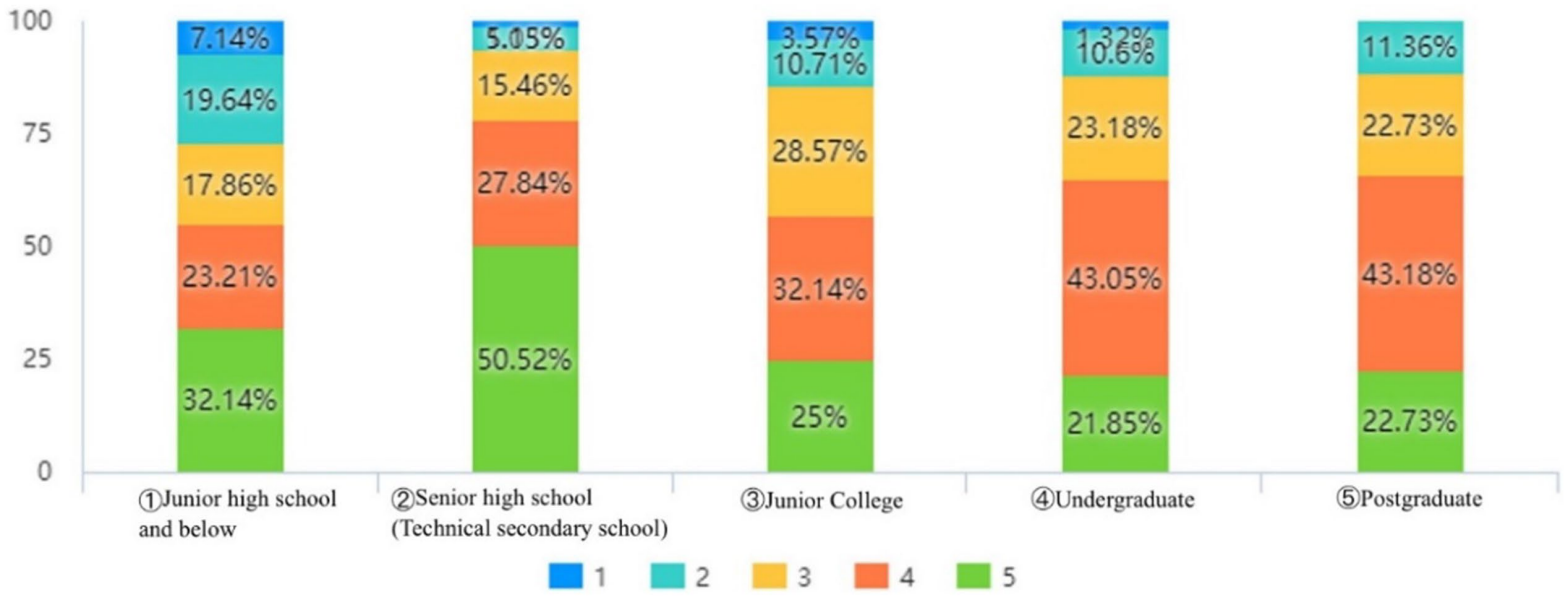
Relationship between education level and satisfaction with COVID-19 prevention and control in the community.

Public’s education level has a considerable impact on community prevention and control satisfaction. The *p*-value of education level and contentment with COVID-19 is 0.004, which is substantially lower than 0.05, indicating that there is a strong association between education level and satisfaction with COVID-19, according to the results of the multifactor analysis. As shown in Figure 4, senior high school (technical secondary school) has the highest level of satisfaction with community prevention and control; undergraduate and postgraduate are extremely satisfied with the degree of similarity.

### 5.3 The manifestations of public panic behavior

Public’s occupation influences their panic consciousness while also influencing the public’s attention to COVID-19 information. The model TSI = 1.719 + 0.009E + 0.117O was obtained after linear regression analysis with education level (E) and occupation (O) as independent variables and how long it takes to pay attention to COVID-19 (TSI) as the dependent variable.

Through the analysis, it is known that the value of the regression coefficient of education level is 0.009 (*t* = 0.117, *p* = 0.907 > 0.05), this result shows that education level does not affect the public’s attention to COVID-19 information. Further analysis shows that the regression coefficient value of occupation is 0.117 (*t* = 4.786, *p* = 0.000 < 0.01), and this result indicates that the public’s different occupations significantly affect how long the public spends paying attention to COVID-19 information every day. A bivariate analysis was conducted with individuals’ “education level” and “occupation” as independent variables and neighborhood location as a covariate. *F* = 1.323, *p* = 0.261 > 0.05 was obtained for individuals’ “education level” and “personal perception of the impact of COVID-19 on life,” and *F* = 1.323, *p* = 0.261 > 0.05 was obtained for individuals’ “occupation” and “personal perception of the impact of COVID-19 on life.” *F* = 2.510, *p* = 0.006 < 0.05 for “personal perception of the impact of COVID-19 on life,” the analysis shows that the public’s occupation has a differential relationship on “personal perception of the impact of COVID-19 on life.” The public’s occupation affects the public’s sense of panic and also influences the behavioral responses adopted by the public during COVID-19.

Some members of the public were apathetic and did not intervene when their friends and family members were unconcerned about COVID-19. According to the research findings, 6.91% of respondents expressly said that they would not take action if their friends and family members did not care about COVID-19. There were also 6.12% of respondents who stated that they were unsure.

**Figure 5:**
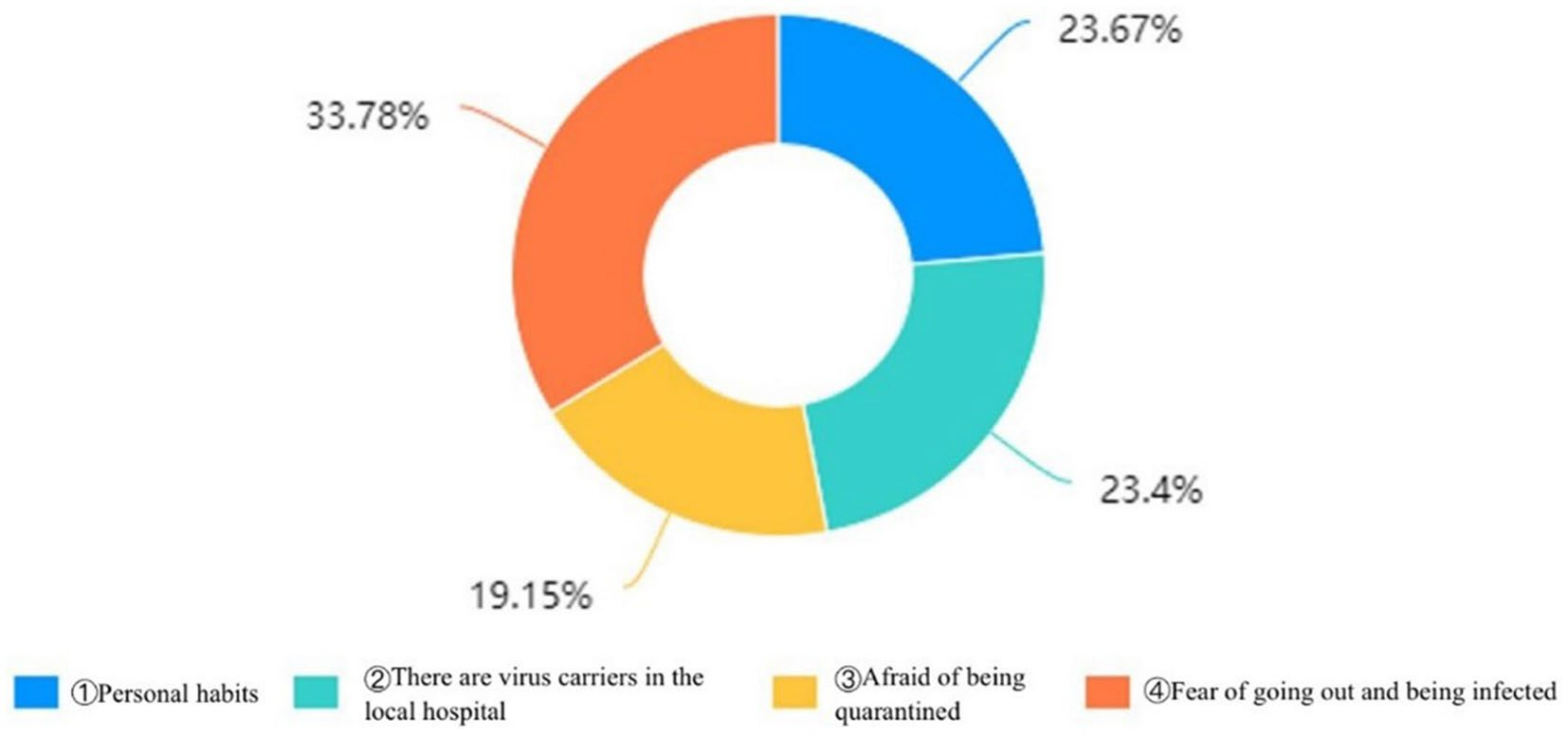
Reasons for not proactively reporting in the first instance.

The main reason why the general people did not report illness symptoms when they recognized them was a fear of increasing their risk of infection. Figure 5 demonstrates that only 23.67% of respondents were initially hesitant to report their illness to groups such as the community due to personal behaviors.

## 6 Conclusion

Synthesizing the above research findings, we have divided the conclusions into three sections based on the KAC and ABC theories: the causes of panic consciousness. The causes of panicky attitudes, and the causes of panic behavior. Through the analysis of research data, we have gained insights into the individual consciousness and attitudes underlying behaviors. By visually presenting the data, we have dissected the panic manifestations behind the data and described them in three dimensions: consciousness, attitude, and behavior.

### 6.1 The causes of panic consciousness

Fear is a natural reaction when facing the pandemic (43). As COVID-19 emerged, it sparked widespread panic among the public, leading to the stigmatization of individuals from Hubei and Wuhan as carriers of the virus, and prompting feelings of fear and concern toward those from these regions. Fear management theory posits that death anxiety is an emotional response characterized by fear or dread, triggered by the proximity of death (44). Given the highly contagious nature of COVID-19, the absence of a specific treatment at the time (45), and the initial shortage of medical resources (46), individuals were faced with a situation fraught with uncertainty. In an environment of incomplete and asymmetric information, personal experiences, and fear, there was an inclination to construe ambiguous threats as immediate dangers (47). This tendency was compounded by the individual’s herd mentality, exacerbating the collective sense of panic.

Emotional bonds serve as a cornerstone for individuals to experience a sense of belonging within a group and to identify with shared values, while also offering the emotional comfort of collective support through group cohesion (48). Panic psychology often manifests as a crisis of social trust (49), and in the midst of the COVID-19 pandemic, individuals found themselves susceptible to mistrust in the social fabric, triggering stress responses (50). This was notably evident in the public’s heightened fear and wary attitudes toward those from Hubei or Wuhan. Confronted with the specter of death, the public tends to prioritize emotional attachments and bonds over broader cultural perspectives, seeking solace in the embrace of groups, particularly family and friends (51). These connections furnish individuals with a robust sense of belonging and identity, making it more conducive for them to dispel feelings of panic under the nurturing influence of emotional bonds.

### 6.2 The causes of panicky attitudes

Panic, as an outward behavioral expression, arises from a group dynamic where individuals, grappling with crises beyond their personal resources, exhibit mass panic (52). The ravaging impact of COVID-19 far exceeded any individual’s capacity to address it single-handedly; it was a force that no one person could conquer alone. In the throes of extreme panic, individuals often find themselves in a state of disarray, with instincts compelling them to fall in line with collective efforts, leading to a wave of conformity. China’s experience during the pandemic bore testament to the fact that isolation was the most potent strategy for curbing the spread of COVID-19 (53). Throughout the period of isolation in China, the vast majority of citizens willingly surrendered certain freedoms, adhering to the government’s management and directives.

The public’s cognitive ability affects their value judgments (54). With diverse educational backgrounds and levels comes a spectrum of cultural literacy and cognitive skills. Individuals who share similar cultural milieus tend to exhibit comparable patterns of logical reasoning, cognitive acumen, and value assessments. The findings from our earlier survey indicate that those with higher levels of education and akin cultural perspectives generally converged in their levels of satisfaction regarding community efforts in COVID-19 prevention and control.

### 6.3 The causes of panic behavior

The public is likely to make value-based decisions that align with their personal expectations and interests. Despite the profound disruption to social order caused by COVID-19, the pandemic’s impact on various sectors of society was magnified due to the distinct nature and content of their work (55). The roles, responsibilities, and obligations of individuals in the context of COVID-19 varied significantly. Consequently, individuals’ focus on COVID-19-related information tended to differ based on their specific occupations, reflecting the diverse ways in which the epidemic affected people’s lives and livelihoods (56).

Members of the public varied in their health awareness, with some demonstrating a notable lack of comprehension regarding the risks posed by COVID-19 (57). Even with the virus’s potent infectious and transmissible nature, many individuals managed to overcome fears and anxiety about mortality through effective psychological adjustments (58). However, the circulation of misinformation that either exaggerated or underestimated the threat of COVID-19 has significantly shaped the public’s view of the epidemic and influenced their behavior (59). At the same time, the level of public safety awareness plays a critical role in epidemic prevention efforts (60). A lack of such awareness often results in underestimating the necessity of health protections. This gap in consciousness is also frequently linked to an individual’s reliance on a mentality, presenting additional challenges in managing the outbreak (61).

The anxiety over potential shortages of essential medical and food resources, the lethal implications of the pandemic, and the inconvenience of enforced quarantine likely engendered widespread distress and panic among the populace (62). As the number of global COVID-19 cases escalated, it was clear that healthcare systems and governmental agencies were underprepared to handle the expansive reach of the outbreak (63). Amidst this, individuals were continually exposed to the risk of infection, and the impact of stay-at-home orders, quarantines, and other epidemic control measures took a toll. This context fostered a climate of skepticism and mistrust within the community, largely rooted in personal considerations of safety and economic stability.

In addition, our findings suggest that the abrupt emergence, unfamiliarity, and high infectivity of COVID-19, coupled with the surrounding uncertainty, played a pivotal role in inciting public panic. The survey data showed a widespread anxiety among the public about the risk of contracting COVID-19, with many perceiving the virus as challenging to manage. While the scarcity of accurate information about the virus certainly contributed to the panic, its impact was not as pronounced as that of uncertainty. Notably, our survey did not establish a link between the transparency of information and the occurrence of public panic. This may be explained by the swift and prioritized response of the Central Committee of the Communist Party of China and the State Council, who underscored the importance of human life and directed all levels of government to release epidemic data promptly through official platforms (64), backed by rigorous accountability measures (65). As a result, the reliability of the information’s timeliness and transparency was well-recognized, and the majority of the public expressed contentment with the government’s handling of information dissemination (66).

## 7 Policy recommendations

Post the COVID-19, it was evident that the general population was grappling with significant levels of worry and stress—a normal physiological response to the crisis at hand (67). It is crucial that these emotional responses are neither ignored nor subjected to criticism. Instead, government agencies must acknowledge their existence, give them due attention, and guide the public accordingly, while also deploying effective methods to channel these sentiments constructively. To bolster the public’s core public health knowledge and response capabilities, it is recommended that various social actors, including schools, communities, and enterprises, facilitate basic health education programs or initiatives. Furthermore, the government should ensure regular public health training and drills to enhance awareness. Educating the public with science-based information during crises can help reduce or eliminate indifference toward those affected by the pandemic within their own circles. To this end, we propose the following five strategies to achieve the outlined goals.

Firstly, it is critical to enhance the development of emotional ties and construct a community that is adept at responding to emergencies. The strong, trust-based relationships among family and friends serve as a cornerstone of support when assistance is needed most (68). By strengthening these emotional links, we can significantly diminish public fear in the wake of natural calamities and bolster collective mental fortitude. In contrast, isolation can result in individuals feeling abandoned and helpless (69). Establishing a united and nurturing community family can provide the necessary emotional support to ease panic and anxiety among members during the outbreak of COVID-19 and similar crises (70).

Secondly, the enhancement of emergency capabilities at the grassroots emergency unit level is of paramount importance. Effective response to public emergencies demands the collaborative involvement of the entire societal fabric (71). It is necessary to establish and maintain professional emergency response teams, to continuously upgrade their response systems and mechanisms, and to prioritize the provision of robust logistical support for emergency operations. In the course of training and drills, the government has a role to play in nurturing a collective ethos among emergency responders, fostering a culture of proactive engagement and commitment to emergency response efforts (72).

Thirdly, it is crucial to enhance the scientific foundation of emergency management practices during extraordinary times. The complexities inherent in government agencies’ exceptional management efforts demand strategies that improve public satisfaction and mitigate the population’s panic. Grassroots leaders should make a concerted effort to heed the insights of community members from varied cultural backgrounds and to reinforce the feedback systems for addressing problems and controversies. A thorough systemic review is necessary to supply the expert panel with comprehensive information, supporting evidence-based policy decisions (73) and allowing for the timely refinement of epidemic control strategies.

Fourthly, it is essential to establish a comprehensive data platform dedicated to crisis information. The disparate work requirements and industry circumstances during the COVID-19 pandemic necessitated unique responses across various professions, with corresponding variations in practitioners’ sensitivity to related epidemic information. Therefore, it is advisable for the government and social entities to collaborate in the creation of a professional-grade information platform, capable of delivering customized information support to individual industries. The dissemination of transparent, open information and data serves as the most effective strategy to counteract rumors and to assuage public concerns (74).

Fifthly, strengthening public trust in times of emergency is a critical component of crisis management. On the one hand, the reporting mechanism for epidemic updates must be made more dynamic and responsive (75). During similar emergencies, it is imperative to streamline the flow of information between hierarchical levels, achieve immediate and honest reporting, maintain transparency, and thus reduce the potential for public panic. On the other hand, the quality of medical and health services in the community must be improved to ensure that the public has access to timely and efficient medical assistance (76).

### 7.1 Limitation of study

Certainly, this study is not without its limitations. Despite the prompt initiation of our survey at the beginning of the outbreak in China, the restricted sample size, a consequence of epidemic control measures, may have compromised our ability to comprehensively depict the public’s panic. Moreover, our examination only provides a snapshot of the initial public reaction, without exploring the evolution of panic across different stages of the epidemic—an aspect that deserves additional scholarly attention. The challenge of accurately gauging public panic during such a time of widespread uncertainty underscores the necessity for more comprehensive research in similar public health crises. As for the 14 underage respondents excluded from our main analysis, their reported lower levels of anxiety and distress, relative to adults, are intriguing. This is likely due to shifts in their routines post-outbreak, including a less rigorous academic load and more personal time, as well as the absence of nearby confirmed cases, which lessened their sense of the pandemic’s immediacy. This finding highlights the importance of focusing on the mental well-being and COVID-19 education of juveniles moving forward.

## Data Availability

The raw data supporting the conclusions of this article will be made available by the authors, without undue reservation.
