# Supplementary material for: The manifestation and causes of public panic in the early stage of COVID-19 in China: a framework based on consciousness-attitude-behavior
Source: Front Public Health. 2024 Dec 3;12:1324382. doi: 10.3389/fpubh.2024.1324382 (PMC11651529; doi:10.3389/fpubh.2024.1324382)
Supplement: Supplementary file 1 [file Data_Sheet_1.docx]

**Appendix: Glossary of terms**

| 1 | 5-point Likert scale | The 5-point Likert scale is a unidimensional scale that allows respondents to indicate their level of agreement or disagreement with a statement. The five response options are usually labeled as follows:  Strongly Disagree - This option represents the strongest disagreement with the statement.  Disagree - This indicates a moderate level of disagreement.  Neither Agree nor Disagree - This represents a neutral stance, indicating no strong inclination towards agreement or disagreement.  Agree - This indicates a moderate level of agreement.  Strongly Agree - This option represents the strongest agreement with the statement.  The Likert scale is considered to be interval in nature, although it does not meet all the criteria for an interval scale due to the subjective nature of the responses. Nonetheless, it is widely used because it is easy to understand and respond to, and it provides a structured way to capture nuanced feedback. Researchers often employ the 5-point Likert scale in questionnaires to assess a range of topics, from customer satisfaction to educational outcomes. The data collected from Likert scales can be analyzed using various statistical methods to uncover patterns, trends, and insights within the responses. |
| --- | --- | --- |
| 2 | Reliability analysis | Reliability analysis in statistics refers to the process of assessing how reliable, or consistent, the measurements from a test or survey are. It is particularly important when the measurements are intended to be used to make inferences about a population or when the results will guide decision-making processes. The reliability of a measurement instrument is often considered a prerequisite for its validity—the extent to which the test measures what it is supposed to measure. |
| 3 | Cronbach's Alpha value | Cronbach's Alpha value is a coefficient of reliability that indicates how closely related a set of items are as a group. It is used to determine the internal consistency of a test or questionnaire, providing an estimate of the extent to which all the items in the test are measuring the same underlying concept or construct. |
| 4 | Mean value of the deleted scale of the item | The mean value of the deleted scale of an item is a statistical measure used to assess the contribution of a specific item to the total score of a scale. It is calculated by first removing the item in question from the scale, then determining the average score of the remaining items for each respondent. This process provides insight into how much the removed item is affecting the overall measurement of the construct. |
| 5 | Deleted scale variance of the item | The deleted scale variance of the term is a statistical measure used to assess the impact of removing a particular term or item from a scale. It is calculated by excluding the scores of that term for all participants and then determining the variance of the remaining terms in the scale. This process provides insight into how much the removed term contributes to the overall variance of the scale. |
| 6 | Corrected item-total correlation | The corrected item-total correlation is a statistical measure that accounts for the intercorrelation among the items in a scale. It is the correlation between a particular item and the total score of the scale, with the total score being computed after excluding the item in question. This correction is necessary because the total score is influenced by the item itself, and without adjustment, the correlation would be artificially high. |
| 7 | Cronbach's alpha if item deleted | Cronbach's alpha if item deleted is a statistical measure that evaluates the internal consistency of a scale once a particular item is no longer part of the scale. It is computed by removing the item in question and then recalculating Cronbach's alpha for the remaining items. This process is repeated for each item in the scale, providing a separate alpha value for each deletion. |
| 8 | Effective Percentage | The effective percentage is a statistical measure that provides a more accurate estimate of the proportion of a population or sample with a specific attribute, once adjustments have been made for potential biases or other influencing factors. It is calculated by applying corrections to the raw data, which might include weighting the responses to account for differences in population demographics or survey response rates. |
| 9 | Cumulative Percentage | The cumulative percentage is a statistical measure that represents the sum of the percentages of a dataset up to a certain point. It is used to show the progressive accumulation of the proportion of a total that is represented by successive data points. |
| 10 | Correlation analysis | Correlation analysis is a technique in statistics that examines the association between variables to determine if they tend to change together and to what extent. The goal is to understand whether a relationship exists, and if so, how strong that relationship is. |
| 11 | χ² (chi-squared) | χ², pronounced as "chi-squared," is a statistical test that measures the difference between observed data and the data that would be expected under a null hypothesis. It is used to determine whether a sample of data is significantly different from what is expected, which can help in making inferences about the population from which the sample is drawn. |
| 12 | ANOVA | Analysis of Variance (ANOVA) is a statistical technique that allows researchers to compare the means of three or more groups to determine if there are statistically significant differences among them. The basic idea is to partition the total variance in the data into variance between groups and variance within groups, and then assess if the between-group variance is significantly greater than what would be expected by chance. |
| 13 | VIF | The Variance Inflation Factor (VIF) is a diagnostic tool used in regression analysis to assess the severity of multicollinearity among the predictor variables. It quantifies how much the variance of an estimated regression coefficient increases due to multicollinearity. |
| 14 | R² (R-squared value) | R², or the coefficient of determination, is a statistical metric that indicates the percentage of the variation in the dependent variable that can be explained by the independent variables in a regression model. essentially, it tells us how much of the data's scatter around the mean can be attributed to the linear relationship between the predictors and the response variable. |
| 15 | Adjusted  R² | Adjusted R² is a statistical measure that adjusts the R² value based on the number of predictors in the regression model, to provide a more accurate assessment of the model's predictive capability. It is used to address the issue of overfitting, where a model may seem to fit the data well simply because it has too many predictors relative to the number of observations. The Adjusted R² is calculated by taking the standard R² value and adjusting it for the degrees of freedom. |
| 16 | D-W value | In statistics, the "D-W value" refers to the Durbin-Watson statistic, which is a test for detecting the presence of autocorrelation in the residuals from a regression analysis. Autocorrelation occurs when the residuals are not independent from each other, which can violate the assumptions of ordinary least squares regression and lead to inefficient and biased estimates. The Durbin-Watson test helps to determine whether the residuals have a positive autocorrelation, negative autocorrelation, or no autocorrelation. |
| 17 | F-test | The F-test is a statistical test used to compare the variances of two populations or to test the significance of a group of regression coefficients in an analysis of variance (ANOVA). It is named after Sir Ronald Fisher, who developed the test. The F-test is based on the F-distribution, which is a probability distribution that arises frequently in the testing of hypotheses about the variances of normally distributed populations. |
| 18 | Two-way analysis of variance | Two-way analysis of variance (ANOVA) is a statistical method used to examine the influence of two independent variables on a single dependent variable. It investigates whether there are statistically significant differences in the means of the dependent variable across different levels of the two independent variables, as well as whether there is an interaction effect between the two independent variables |
| 19 | Square sum | In statistics, the term "square sum" typically refers to the sum of squared deviations or the sum of squared values. This concept is used in various statistical calculations, such as the calculation of variance, standard deviation, and in regression analysis. The square sum is particularly important because squaring the deviations or values makes all the terms positive and emphasizes larger deviations, which is useful for measuring dispersion and constructing statistical models. |
| 20 | df | In statistics, "df" stands for degrees of freedom, which is a critical concept in hypothesis testing, confidence intervals, and the calculation of certain statistical indices. Degrees of freedom refer to the number of values in a calculation that are free to vary without constraint. It is an important parameter that determines the shape of probability distributions like the t-distribution and the F-distribution, which are used in various statistical tests. |
